# Evaluation of TPGU using entropy - improved TOPSIS - GRA method in China

**DOI:** 10.1371/journal.pone.0260974

**Published:** 2022-01-21

**Authors:** Hua Dong, Kun Yang, Guoqing Bai

**Affiliations:** 1 School of Economics and Management, North China Electric Power University, Beijing, China; 2 School of Economics and Management, Beijing University of Technology, Beijing, China; Gonbad Kavous University, ISLAMIC REPUBLIC OF IRAN

## Abstract

China is still one of the countries dominated by thermal power generation. In order to generate more efficient, stable and clean power, it is necessary to evaluate thermal power generation units (TPGU). Firstly, a comprehensive evaluation index system for TPGU with 20 secondary indicators was established from four aspects: reliability indicators, economic indicators, technical supervision indicators, and major operating indicators. Secondly, the entropy weight method can be used to calculate the weight of each second-level index. Mahalanobis Distance improved Technique for Order Preference by Similarity to an Ideal Solution (TOPSIS) method is coupled with the Grey Relational Analysis (GRA), and the comprehensive evaluation values of 5 units (600MW) are respectively 0.4516, 0.5247, 0.3551, 0.5589 and 0.6168 from both vertical and horizontal dimensions. Finally, by comparing and analyzing this method with the above research methods, it is found that the results obtained by this method which re-establishes the coordinate system based on the data set are more accurate. In addition, this method can effectively evaluate the operation of TPGU, which is of great significance for cleaner production while generating electricity. In conclusion, some suggestions on clean production of TPGU are put forward, and the innovation points and limitations of this paper are pointed out.

## 1. Introduction

At present, the proportion of various types of new energy power generation is constantly increasing. However, due to China’s energy structure, coal-fired thermal power generation is still the main form. The BP World Energy Outlook (2019), released by British Petroleum, points out that China’s energy structure is continuously evolving, with coal’s share falling from 60% in 2017 to 35% in 2040 [1]. In addition, with the vigorous implementation of China’s electricity reform, the continuous reduction of electricity price, and the increasingly stringent policies on energy conservation and emission reduction, power plants must adopt high-efficiency, stable, energy-saving and environment-friendly TPGU to meet the challenge. The production and operation of thermal power generation enterprises will face unprecedented tremendous pressure. Reasonable comprehensive evaluation of power plant units is the guarantee for consumption reduction and optimized operation [2].

The realization of the goal of "carbon neutral" requires a new low-carbon development transformation strategy [3], which will force China’s energy transformation and make it gradually get rid of the dependence on fossil fuels [4], which will make the thermal power industry face great challenges and difficulties. China’s future economy will still maintain a high growth rate [5]. China’s energy endowment is more coal, lean oil, less gas, the current power generation structure is mainly thermal power [6, 7]. Under the goal of "carbon neutral", China’s non-petrochemical power generation will account for more than 85% of the total power generation by 2050, while the proportion of coal will be reduced to less than 5% [8]. Therefore, it is an arduous task for China’s power structure to transform into a low-carbon power structure and finally achieve net zero.

Therefore, the establishment of a scientific and objective evaluation method for TPGU is not only of great significance to the optimization of thermal power plant energy conservation, but also plays an important role in promoting the sound development of TPGU, social stability and even the development of the entire national economy.

As one of China’s main power generation methods, thermal power generation will not be replaced by other power generation methods in the short term. Therefore, when the pollution caused by thermal power generation is lower than a certain standard and the power generation efficiency is high enough, this kind of power generation method can also be regarded as "clean energy". China and the world have made great efforts to implement the green and low-carbon concepts of the Paris Agreement [9–11], focusing on themes such as thermal power generation and TPGU. China remains the world’s largest emitter of carbon dioxide, mainly due to the high proportion of coal-fired thermal power [12, 13]. The flexibility of TPGU [14–17], SO_2_ emissions [18] and other indicators can only be used as a kind of technical indicators or TPGU improvement targets, which cannot be used to comprehensively evaluate TPGU.

In recent years, China’s installed capacity of renewable energy has increased significantly. In order to reduce the proportion of thermal power plants, TPGU should be evaluated and low-rated power generation units should be eliminated in an orderly manner. However, there is still no effective and accurate evaluation criteria and methods to eliminate them. The contribution of this study is to propose a new fusion method for the orderly elimination of TPGU. This method mainly consists of Entropy, Improved TOPSIS and GRA method. Entropy is used to determine the index weight of evaluating TPGU. After data processing, the correlation between indexes can be clearly reflected, the calculation process is simple and easy to operate, the objectivity is strong, and the result is feasible. TOPSIS-GRA can evaluate TPGU from both vertical and horizontal dimensions. TOPSIS method calculates the proximity between TPGU and positive and negative ideal solutions (the best and worst TPGU in each index), and then sorts all TPGU, with less consideration on correlation. GRA determines the TPGU ranking by calculating the correlation degree between the comparison TPGU and the reference TPGU, which lacks consideration of the whole aspect. TOPSIS-GRA method organically integrates the advantages of TOPSIS method and GRA in entirety analysis and correlation analysis, so TOPSIS-GRA method has strong applicability and objectivity in the evaluation of TPGU. In particular, the traditional Euclidean distance is replaced by Mahalanobis Distance in TOPSIS method. This method has the characteristics of establishing coordinate system based on data, which is different from Euclidean distance. The results obtained by the fusion method are more accurate, so the fusion method is an effective method to eliminate TPGU.

The research structure of this paper is as follows. Section 2 is about the evaluation of TPGU, TOPSIS and TOPSIS-GRA related literature review. Section 3 establishes a comprehensive evaluation index system for TPGU. Section 4 introduces the basic theory and model mechanism. Section 5 describes the computational steps of the model. Section 6 conducts a comprehensive evaluation of five generating sets according to Section 5, and the evaluation results obtained are consistent with the actual results of the competition. In Section 7, the results of this paper are compared with the other two methods and discussed and analyzed. Section 8 summarizes the contribution and deficiency of this paper to cleaner production and points out the direction of future comprehensive evaluation of TPGU.

## 2. Literature review

The study on efficiency evaluation of TPGU is of great significance for reducing environmental pollution and gradually phasing out TPGU. [12, 19] studies the efficiency of coal-fired power plants, analyzes the main reasons of low efficiency of coal-fired power plants and evaluates power plants in 30 provinces, and concludes that the efficiency of large power plants can be higher than that of small power plants. [20] used fuzzy technology to solve the problem of incomplete information in the evaluation process, and evaluated the efficiency, installation cost, carbon dioxide emission and other aspects of the thermal power plant. [21] proposed several mathematical approaches to evaluate performance of Iranian thermal power plants. In addition, [22] using the AHP (analytic hierarchy process) evaluated and prioritized some parts of a thermal power plant.

Other multi-criteria decision making (MCDM) methods can also be used to compare and sort schemes. COPRAS (COmplex PRoportional Assessment) [23] can effectively integrate the importance and effectiveness of evaluation indicators when evaluating the program. And developed Fuzzy COPRAS [24, 25], hesitant fuzzy linguistic COPRAS [26] and other methods. WASPAS (Weighted Aggregates Sum Product Assessment) [27, 28] is a multi-attribute decision making method based on Weighted Sum model and Weighted Product model. Compared with single method, WASPAS method can improve the accuracy of aggregation and make the decision-making process more accurate. SECA (Simultaneous Evaluation of Criteria and Alternatives) [29, 30] do not need to independently determine the objective weight of each attribute and then evaluate it through multi-attribute decision making method. The objective of this method is to determine the overall score of each scheme and the objective weight of each attribute simultaneously. MEREC (MEthod based on the Removal Effects of Criteria) [31, 32] uses removal effects of each criterion on the aggregate performance of alternatives for calculating criteria weights. In the proposed method, a criterion has a greater weight when its removal leads to more effects on alternatives’ aggregate performances. Besides weighting e ach criterion, this perspective may help decision-makers to exclude some criteria from the decision-making process. EDAS (Evaluation based on Distance from Average Solution) [33] changes the evaluation standard from the extreme optimal and worst solution to the average solution. The compromise solution can improve the decision-making efficiency of the decision-making group when there are large differences in a variety of schemes. And developed interval fuzzy EDAS [34], 2-type fuzzy EDAS [35] and other methods. TOPSIS method has clear logic and stable results, and its approach degree can better summarize group decision-making information. The data distribution and sample content is not strictly limited, data calculation is simple. This advantage is not available in the above methods, so this paper selects TOPSIS method to evaluate TPGU.

In recent years, the combined application of TOPSIS-GRA has been applied in enterprises, medical treatment, electric power engineering and other fields. [36, 37] applied this method to supplier selection as a multi-attribute group decision making method. [38] used this method to evaluate engine emissions. It is also used in medical industry that evaluated Risk Priority Number evaluation for a medical device prototype [39]. In the agricultural field, there are also examples of applying this method to green supplier selection in the agri-food industry [40]. [41] provided a novel risk priority approach for failure mode and effect analysis and is applied to a practical healthcare risk analysis case. [2] used entropy weight method to determine the weight of indicators. Then, TOPSIS-GRA method was used to comprehensively evaluate the TPGU, and the TPGU were sorted from the vertical and horizontal dimensions.

The above method ignores the correlation between indexes in the application of TOPSIS method, that is, the change of one index greatly affects other indexes, which may be positive correlation or negative correlation. Euclidean distance should not be used when the correlation between indexes is large. It can be seen that the TOPSIS GRA method has been applied very well in various fields and is an effective evaluation method. However, it should not be used when the correlation between indicators is large. Therefore, this method needs to be improved before use.

The above method ignores the correlation between indexes in the application of TOPSIS method, that is, the change of one index greatly affects other indexes, which may be positive correlation or negative correlation. Euclidean distance should not be used when the correlation between indexes is large. It can be seen that the TOPSIS GRA method has been applied very well in various fields and is an effective evaluation method. However, it should not be used when the correlation between indicators is large. Therefore, this method needs to be improved before use.

On the one hand, previous studies focused on the combination of methods. Because no one method is perfect, so take the advantages of each method, combine two or three methods to make the final result more ideal. But it doesn’t actually change the final order, it actually makes the calculation more complicated. On the other hand, the method is improved by fuzzifying. These methods to minimize errors caused by factors such as incomplete information and human cognitive limitations so as to make the results more accurate. Different from previous studies, this paper not only integrates the three methods, improves the methods, and even changes the coordinate system of data. That is to say, previous studies did not establish coordinate system based on data, but the method proposed in this paper changed the direction of coordinate system based on the characteristics of data, as detailed in Basic Theory.

## 3. Comprehensive evaluation index system of TPGU

**Fig 1:**
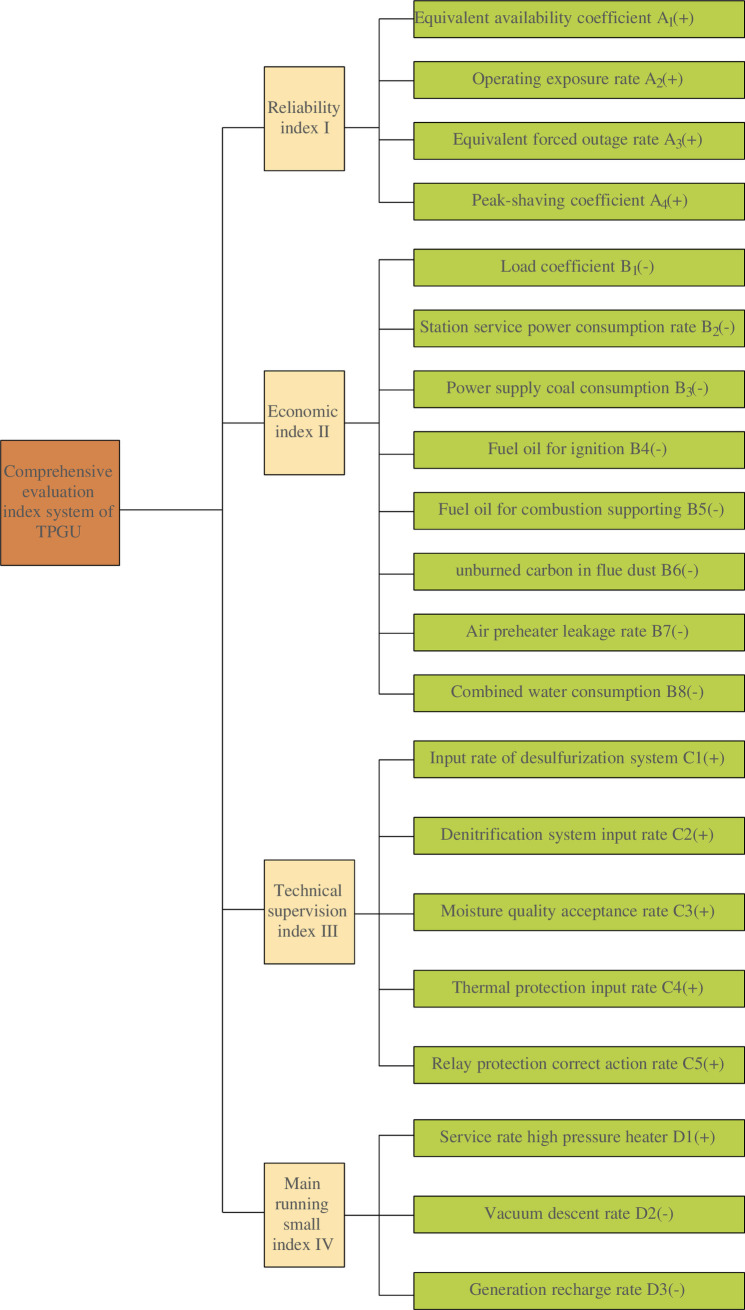
Comprehensive evaluation index system of TPGU.

This paper takes the competition index of China Electricity Council (CEC) as a reference. Based on the actual situation of the data reported by thermal power plants and the opinions of the evaluation expert group, the indicators were selected into four categories of first-level indexes, including reliability index, economic index, technical supervision index, main operating little index, and 20 sub-level indexes. The classification of specific indexes is shown in Fig 1. Among them, the higher the value is, the better the positive index is, which is represented by "+"; On the contrary, the smaller the value is, the better the reverse index is, which is represented by "-".

### 3.1. Reliability index

The reliability of power generation system is to evaluate the capacity of all generating units in unified grid-connected operation to meet the power and power demand of the power system load according to the acceptable standard and the desired quantity [42]. It includes four aspects: A1-A4.

A1: Equivalent availability factor(+) (EAF)

Based on the operation and maintenance experience of TPGU, the maintenance interval is determined according to the operation reliability index of units. EAF is determined by the following formula:

EAF = (the unit operating hours-the unit reduced output equivalent shutdown hours)/the unit’s statistical hours

There are three factors affecting EAF, namely, planned shutdown of units (including major, minor repairs and planned temporary maintenance), unplanned shutdown and unplanned reduction of unit output [43].

A2: Operating exposure rate(+) (EXR) and A3: Equivalent forced outage rate (-) (EFOR).

Reliability is not only related to the safety and stability of power grid, but also reflects the management and technical level of power production enterprises. A large amount of coal and fuel will be consumed when the generator sets start and stop due to malfunction. Therefore, the two monitoring indexes of operation exposure rate and equivalent forced shutdown rate are increased to better monitor the operation and management of the power plant. The operating exposure rate is the proportion of the operating hours in the available hours, which reflects the actual operating condition of the generator set. The higher the running exposure rate, the closer it is to the number of available hours. The equivalent forced shutdown rate is the forced shutdown rate of units after reducing the influence of output [44, 45].

A4: Peak-shaving coefficient (+) (PSC)

PSC = (Leverage hours * available hours)/(running hours * equivalent available hours)

The calculation of the comprehensive peak adjustment coefficient of TPGU is complex, and it cannot fully reflect the peak adjustment status of the system [46].

### 3.2. Economic index

The economic performance indexes of power plant operation mainly include standard coal consumption rate (quantity) and power utilization rate. Due to the improvement of technology, the maximum efficiency of the power plant has reached more than 40%, and the thermal power plant can reach 60%. Therefore, most power plants measure the operation economy of the TPGU by the standard coal consumption rate and the power utilization rate. Combined with the characteristics of the TPGU, five indexes are added, including seven aspects of B1-B8.

B1: Load coefficient (+)

Also known as the average utilization rate of power generation equipment, it is an index reflecting the utilization degree of power generation equipment [47].

B2: Station service power consumption rate (-)

That is, the percentage of factory variable power consumption and power generation per unit time. Reduce the power consumption rate and power generation cost of thermal power plants to the maximum extent, and improve the market competitiveness of power plants.

B3: Power supply coal consumption (-)

Power supply coal consumption is one of the most important technical and economic indicators of power plants, which comprehensively reflects the technical management level and economic effect of power plants. With the continuous deepening of energy saving and consumption reduction work, reducing Power supply coal consumption is the most important work of coal-fired power generation enterprises [48].

B4: Fuel oil for ignition (-)

Plasma ignition technology is a high-efficiency and energy-saving technology. Its promotion can greatly reduce the ignition and combustion-supporting oil of thermal power plants, realize the replacement of oil by coal, save a lot of combustion-supporting oil, and create significant economic benefits for power plants [49].

B5: Fuel oil for combustion supporting (-)

The adoption of micro-oil ignition technology can save a large amount of combustion-supporting oil and reduce the cost [50].

B6: unburned carbon in flue dust (-)

The unburned carbon in flue dust is an important index of TPGU, which can directly reflect the boiler efficiency of the TPGU. Real-time and accurate monitoring of the unburned carbon in flue dust is helpful for operators to adjust the operation mode at any time and control the carbon content of fly ash in the optimal range. In this way, the combustion degree can be maximized and the operation level of the unit can be improved [51].

B7: Air preheater leakage rate (-) (APLR)

APLR = the mass flow of air leaking into the flue gas side of the air preheater/the mass flow of flue gas entering the air preheater

Air leakage from the air preheater will reduce the thermal efficiency of the TPGU. At the same time, the power consumption of ventilation machinery is increased, which increases the coal consumption of power generation and power supply in power plants [52].

B8: Comprehensive water consumption rate (-) (CWCR)

CWCR = the amount of water used for power generation/the amount of power generation

The less fresh water a power plant consumes, the better. Reducing the water consumption rate includes strengthening the operation and management of circulating water, formulating chemical water-saving measures, strengthening the water use management of flushing ash water, and strengthening the recycling and utilization of industrial water, etc. [53].

### 3.3. Technical supervision index

This paper focuses on the high efficiency, energy saving, environmental protection and safety performance of the TPGU. Therefore, the environmental protection supervision index in technical supervision is the main one, supplemented by relay protection supervision, thermal engineering supervision, and chemical supervision. C1-C5 main evaluation indexes of the TPGU are selected.

C1: Desulfurization system input rate (+)

The operation of the desulfurization system consumes a lot of electric energy and materials. However, it cannot create profitable products for power generation enterprises. The power plant is not active in the desulfurization system. With the introduction of more stringent environmental protection policies in China, the operation status of desulfurization system is directly related to the on-grid electricity price. Only stable and reliable operation can bring economic benefits, otherwise, power generation enterprises will be subject to economic penalties [54].

C2: Denitrification system input rate (+)

The installation and operation of the denitrification system are likely to cause problems such as the corrosion of the rear air preheater at the tail, ash blocking and the increase of differential pressure. When the boiler is not undergoing technical transformation of denitrification system, to increase the inlet temperature of the denitrification reactor through operation optimization and adjustmentis is the most practical and effective way to increase the denitrification system input rate of coal-fired units [55].

C3: Steam water quality acceptance rate (+) (SWQAR)

SWQAR = the steam water quality acceptance times/the total sampling times (%)

Due to the small difference in the qualified rate index value of steam water quality of each power plant and the small value differentiation, it mainly relies on other indicators for judgment [56].

C4: Utilization factor of thermal protection system (+) (UFTPS)

UFTPS = the number of sets of thermal protection system invested/the total number of sets of thermal protection system designed

During the trial production period or after overhaul, the accuracy rate of thermal instruments and the input rate of thermal protection shall reach more than 90% [57].

C5: Relay protection correct action rate (+) (RPCAR)

RPCAR = the correct operation times of relay protection/the total operation times of relay protection

In order to improve the accuracy of relay protection actions, it is necessary to clarify the focus of relay protection, rationally config and transform relay protection devices, and improve the professional quality and management level [58].

### 3.4. Main running small index

The small operation index is the guarantee of energy saving and safe production. The index size is directly related to the operation optimization and economic benefits of the power plant. In the operation process of the unit, the influence of the initial and final parameters on the unit power is generally determined by managing the small indexes of the operation.

D1: Service rate of high pressure heater (+) (HPHSR)

HPHSR = the operating hours of high-pressure heaters/the corresponding operating hours of TPGU

The frequent leakage of the high heater, the easy fracture of the high heater electric heads and the improper operation method are the main reasons for the low input rate of the high heater [59].

D2: Vacuum descent rate (-)

During the normal operation of the unit, a vacuum tightness test should be carried out at intervals. The vacuum descent rate is indicated by the Vacuum descent rate. Through experiments, it can be concluded that the vacuum tightness changes caused by the vacuum leakage amount of the unit, so as to regularly analyze the operation status of the equipment.

D3: Make-up water rate of power generation (-) (MUWR)

MUWR = make-up amount of desalinated water/the amount of boiler steam in the calculation period

The high make-up water rate of power generation is mainly caused by the large consumption of boiler ash blower, large discharge of boiler sewage, internal leakage of valve and hydrophobic discharge [60].

## 4. Basic theory

### 4.1. Mahalanobis distance improved TOPSIS method

Mahalanobis Distance [61, 62] was proposed by the Indian statistician (P. C. Mahalanobis) in 1936, which represents the covariance Distance of data. It is an effective method to calculate the similarity between two unknown sample sets. Unlike Euclidean distance, Mahalanobis Distance takes into account the connection between various characteristics (for example, there is a certain correlation between height and weight). Mahalanobis Distance is not affected by dimension. Mahalanobis Distance between two points has nothing to do with the measurement unit of the original data and has the advantage of eliminating the correlation interference between variables.

In TOPSIS method, the European distance is appropriate when the correlation coefficient between indexes is not large, but when the correlation coefficient is very close to 1, the error result will appear in the Euclidean distance. The Mahalanobis Distance is actually seeking the degree of difference between a single sample data and the whole data set. In other words, a new coordinate system different from the Euclidean distance is re-established on the basis of the data set, and a certain degree of deviation from the whole is calculated in the new coordinate system.

**Fig 2:**
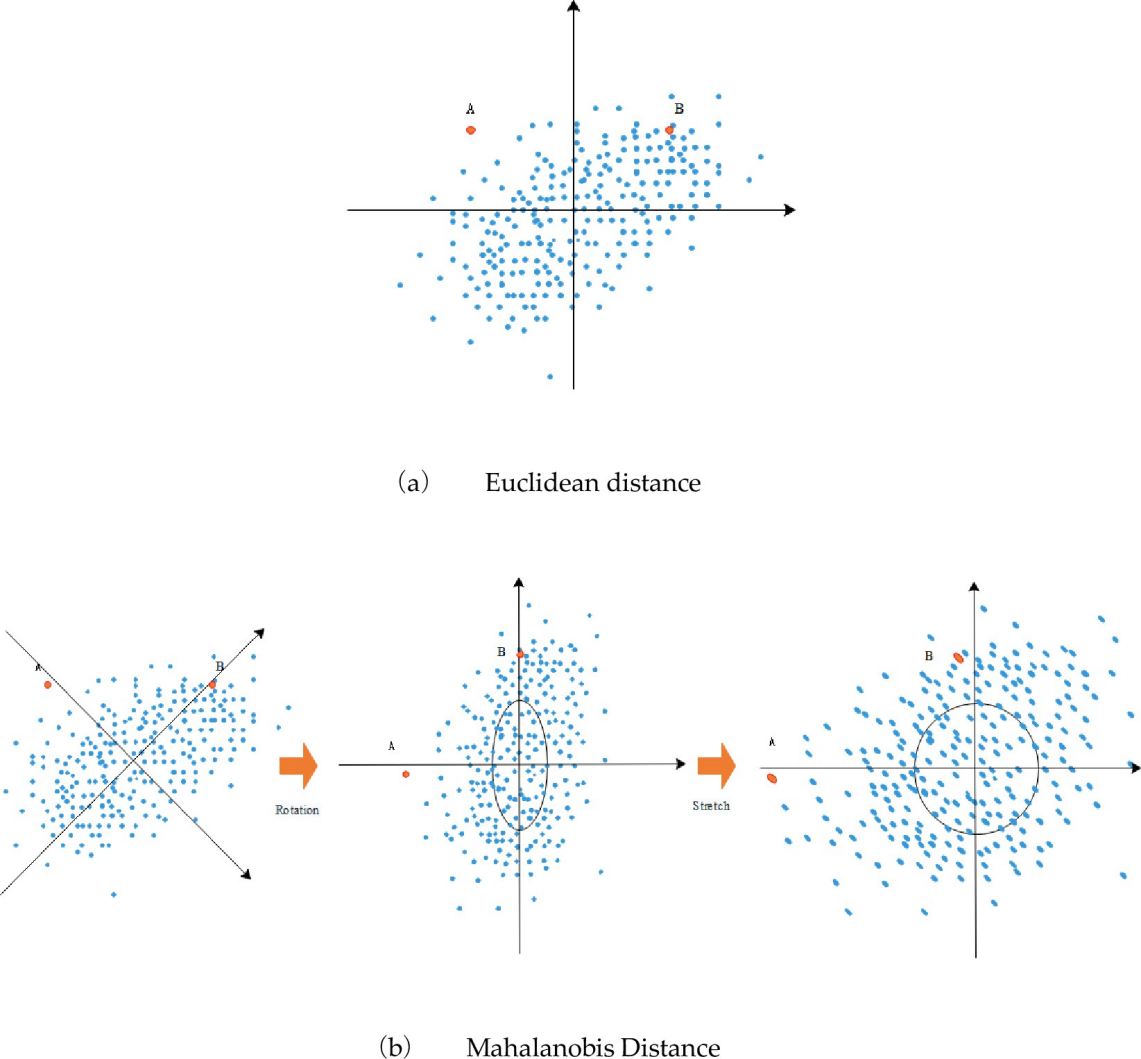
Diagram of Euclidean distance and Mahalanobis distance. (a) Euclidean distance. (b) Mahalanobis distance.

In Fig 2A, the Euclidean distances from A and B to the origin are equal, which means that A and B are equally important relative to the overall sample. For the same overall sample, to calculate the Mahalanobis Distance, we need to re-establish the coordinate system (b) in the overall sample. The origin is the average of the samples, and the vertical axis of the coordinate is drawn along the direction of maximum data variance. Then we rotate the coordinate axis and stretch it. The result is that point B is closer to the overall sample, and obviously, (b) is more in line with the facts. In the TOPSIS method, the Euclidean distance of the sample data deviating from the positive and negative ideal solutions is converted to the calculation of the Mahalanobis Distance.

This method has the following three advantages:

- The calculation is simple, practical and operable, and easy to promote;
- The mathematical method is non-statistical, and there is no requirement for the size and distribution of the sample;
- There will be no inconsistency between the quantitative results of the relational degree and the qualitative analysis.

### 4.2. Mechanism of the model

**Fig 3:**
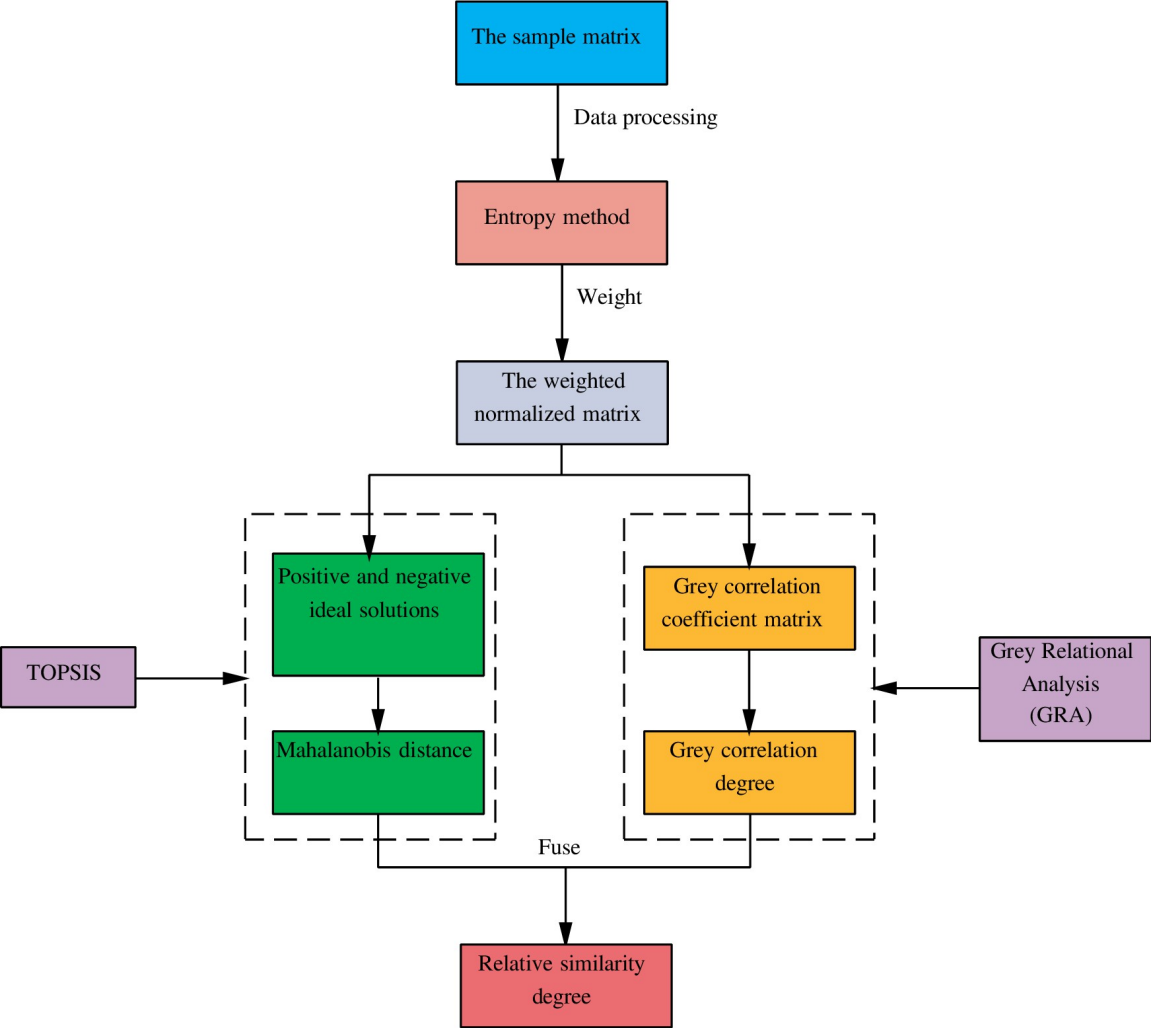
The mechanism of improved TOPSIS and grey relational analysis model.

Firstly, the data of 5 TPGU are set up as sample matrix and data processing is performed. Secondly, the entropy weight method is used to weight each index, so as to obtain a weighted standardized matrix. Thirdly, the Mahalanobis Distance improved TOPSIS method can obtain the Mahalanobis Distance from each unit to the positive and negative ideal solutions of each index. Then, the grey relational analysis method is used to obtain the correlation degree of each unit to the positive and negative ideal solutions of each index. Finally, the Mahalanobis Distance and the grey relational degree are merged to determine the relative closeness, and the final sorting result of the thermal power unit is obtained. As shown in Fig 3 below.

## 5. Comprehensive evaluation model

### 5.1. Establishment of the sample matrix

Step 1: Establishment of index matrix

Set *A* = {*A*_1_, *A*_2_,⋯,*A*_*m*_} as the sample set of TPGU, and *B* = {*B*_1_, *B*_2_,⋯,*B*_*n*_} as the evaluation index set. Constitute the index evaluation matrix *X* = (*x*)_*m*×*n*_, where *x*_*ij*_ is the *j* evaluation index of the *i* unit, *i* = 1, 2,⋯, *m*, *j* = 1, 2,⋯, *n*.


$$X={\left(x\right)}_{m\times n}=\begin{array}{c} A_{1}\\ A_{2}\\ \vdots \\ A_{m} \end{array}\begin{array}{cccc} B_{1} & B_{2} & \cdots & B_{n} \end{array}\left[\begin{array}{cccc} x_{11} & x_{12} & \cdots & x_{1n}\\ x_{21} & x_{22} & \cdots & x_{2n}\\ \vdots & \vdots & & \vdots \\ x_{m1} & x_{m2} & \cdots & x_{mn} \end{array}\right]$$ (1)

Step 2: Data processing

The unit and magnitude of each indicator are different, and the data is normalized to obtain a dimensionless decision matrix Y = (y_*ij*_)m×n.

For efficiency indexes, the higher the value, the better the TPGU performance is, standardization:

$$y_{ij}=\frac{x_{ij}-\underset{i}{\min}\{x_{ij}\}}{\underset{i}{\max}\{x_{ij}\}-\underset{i}{\min}\{x_{ij}\}}$$ (2)

For cost indexes, the lower the value, the better the TPGU performance is, standardization:

$$y_{ij}=\frac{\underset{i}{\max}\{x_{ij}\}-x_{ij}}{\underset{i}{\max}\{x_{ij}\}-\underset{i}{\min}\{x_{ij}\}}$$ (3)

### 5.2. Determine the weight of each index

Step 3: Entropy value

According to (1), the entropy value of index *j* can be obtained:

$$E_{j}=-\frac{1}{\text{ln}\;m}\sum_{i=1}^{m}y_{ij}\;\text{ln}(y_{ij}),j\in n,i\in m$$ (4)

Where, 0 ⩽ *E*_*j*_ ⩽ 1, 0 ⩽ *y*_*ij*_ ⩽ 1 and $\sum_{i}^{m}y_{ij}=1$. When *y*_*ij*_ = 0, set *y*_*ij*_ ln(*y*_*ij*_) = 0.

Step 4: For the weight

Weight is:

$$w_{j}=\frac{1-E_{j}}{\sum_{j=1}^{n}\left(1-E_{j}\right)}$$ (5)

Where, 1—*E*_*j*_ is the index utility value, and the weight vector is obtained:

$$W={\left(w_{1},w_{2},\cdots ,w_{n}\right)}^{T}$$ (6)

Step 5: Weighted standardized index matrix

The index weight is multiplied by each index of the TPGU vector after normalization, to obtain a weighted standardized index matrix *Z* = (*z*_*ij*_)_*m*×*n*_:

$$Z={\left(z_{ij}\right)}_{m\times n}={\left(w_{j}y_{ij}\right)}_{m\times n}$$ (7)

### 5.3. Mahalanobis distance improvement TOPSIS

Step 6: Ask for the positive and negative ideal solutions. Since the data has been standardized in Step 2, the attribute difference caused by the cost and benefit indicators is eliminated, and the solution can be solved directly.


$$Z_{0}^{+}=(\underset{1\leq i\leq m}{\max}z_{ij}\left|j\in j^{+}\right|,\underset{1\leq i\leq m}{\min}z_{ij}\left|j\in j^{-}\right|)=(z_{1}^{+},z_{2}^{+},\cdots ,z_{m}^{+})$$ (8)

$$Z_{0}^{-}=(\underset{1\leq i\leq m}{\min}z_{ij}\left|j\in j^{+}\right|,\underset{1\leq i\leq m}{\max}z_{ij}\left|j\in j^{-}\right|)=(z_{1}^{-},z_{2}^{-},\cdots ,z_{m}^{-})$$ (9)

Step 7: Obtain the Mahalanobis Distance from each unit to the positive and negative ideal solutions. Assume that the coordinates of the positive ideal solutions $Z_{0}^{+}=(z_{1}^{+},z_{2}^{+},\cdots ,z_{m}^{+})$ and negative ideal solutions $Z_{0}^{-}=(z_{1}^{-},z_{2}^{-},\cdots ,z_{m}^{-})$ are fixed, while, *A*_*i*_ ={*x*_*i*1_, *x*_*i*2_,⋯,*x*_*in*_} which represents the index space coordinates corresponding to the i unit is changed. Where, Σ^-1^ is the inverse of the covariance matrix Σ of *n* attribute variables.


$$d(z_{i},Z_{0}^{+})=\sqrt{(z_{i}-Z_{0}^{+})\Sigma^{-1}{\left(z_{i}-Z_{0}^{+}\right)}^{T}}$$ (10)

$$d(z_{i},Z_{0}^{-})=\sqrt{(z_{i}-Z_{0}^{-})\Sigma^{-1}{\left(z_{i}-Z_{0}^{-}\right)}^{T}}$$ (11)

### 5.4. Grey relational degree

Step 8: According to the weighted standardized index matrix (7), the grey relational coefficient of the *i* unit sample on the *j* index of positive and negative ideal solutions is determined:

$$r_{ij}^{+}=\frac{\underset{i}{\min}\underset{j}{\min}\left|z_{j}^{+}-z_{ij}\right|+\rho \underset{i}{\max}\underset{j}{\max}\left|z_{j}^{+}-z_{ij}\right|}{\left|z_{j}^{+}-z_{ij}\right|+\rho \underset{i}{\max}\underset{j}{\max}\left|z_{j}^{+}-z_{ij}\right|}$$ (12)

$$r_{ij}^{-}=\frac{\underset{i}{\min}\underset{j}{\min}\left|z_{j}^{-}-z_{ij}\right|+\rho \underset{i}{\max}\underset{j}{\max}\left|z_{j}^{-}-z_{ij}\right|}{\left|z_{j}^{-}-z_{ij}\right|+\rho \underset{i}{\max}\underset{j}{\max}\left|z_{j}^{-}-z_{ij}\right|}$$ (13)

The grey relational coefficient matrices $R^{+}={\left(r_{ij}^{+}\right)}_{m\times n}$ and $R^{-}={\left(r_{ij}^{-}\right)}_{m\times n}$ are obtained. Where, *ρ* is the discrimination coefficient, *ρ* ϵ [0,1], and usually *ρ* = 0.5[63].

Step 9: Determine the relational degree

According to (12) and (13), the relational degree between the *i* unit sample and the positive and negative ideal solutions is determined:

$$s_{i}^{+}=\frac{1}{n}\sum_{j=1}^{n}r_{ij}^{+},i=1,2,\cdots ,m$$ (14)

$$s_{i}^{-}=\frac{1}{n}\sum_{j=1}^{n}r_{ij}^{-},i=1,2,\cdots ,m$$ (15)

### 5.5. Fusion of Mahalanobis distance and GRA

Step 10: Dimensionless treatment of Mahalanobis Distance and grey relational degree

$$D_{i}^{+}=\frac{d_{i}^{+}}{\underset{i}{\max}d_{i}^{+}}$$ (16)

$$D_{i}^{-}=\frac{d_{i}^{-}}{\underset{i}{\max}d_{i}^{-}}$$ (17)

$$S_{i}^{+}=\frac{s_{i}^{+}}{\underset{i}{\max}s_{i}^{+}}$$ (18)

$$S_{i}^{-}=\frac{s_{i}^{-}}{\underset{i}{\max}s_{i}^{-}}$$ (19)

Step 11: To fuse dimensionless Mahalanobis Distance and grey relational degree.

The larger the values of $D_{i}^{-}$ and $S_{i}^{+}$, the closer the sample is to the ideal solution. On the contrary, the larger the $D_{i}^{+}$ and $S_{i}^{-}$ values are, the more the sample deviates from the ideal solution, and we can obtain that:

$$E_{i}^{+}=\alpha D_{i}^{-}+\beta S_{i}^{+},i=1,2,\cdots ,m$$ (20)

$$E_{i}^{-}=\alpha D_{i}^{+}+\beta S_{i}^{-},i=1,2,\cdots ,m$$ (21)

*α*, *β* respectively reflect the deviation degree of evaluator to the position and shape. Among them, *α*, *β* ϵ [0,1], generally take *α* = *β* = 0.5[64]. $E_{i}^{+}$ comprehensively reflects the degree of approximation between the sample and the ideal solution, and the larger the value, the better; $E_{i}^{-}$ is the opposite.

Step 12: Relative closeness coefficient

The relative closeness coefficient of the *i* unit sample is:

$$\gamma_{i}=\frac{E_{i}^{+}}{E_{i}^{+}+E_{i}^{-}},\;i=1,2,\cdots ,m$$ (22)

## 6. Case study

### 6.1. Data sample

In this paper, five 600MW subcritical TPGU in China’s national thermal power unit competition are selected as the evaluation object, and the code of the units is a-d [65]. The data were all from the annual TPGU operation data reported to China Electricity Council (CEC) by the various power generation groups. According to the index system established in Section 3, the selected data is shown in S1–S4 Tables.

Step1: The sample matrix X is established according to the data in the table, where the element in set *A* = {*A*_1_, *A*_2_,⋯,*A*_*m*_} represents the sample data of each unit, and set *B* = {*B*_1_, *B*_2_,⋯,*B*_*n*_} represents that there are n indexes in B.

### 6.2. Sample calculation

Step 2–4: According to Eqs (1)–(6), the weight values of the first and second indexes are obtained as shown in the following Table 1.

**Table 1:** The weight of TPGU comprehensive evaluation index.

	First-level index	Weight	Second-level index	Weight
Evaluation indexes of unit Comprehensive	Reliability indexes I	0.1560	Equivalent availability coefficient A1(+)	0.0279
			Operating exposure rate A2(+)	0.0478
			Equivalent forced outage rate A3(+)	0.0393
			Peak-shaving coefficient A4(+)	0.0410
	Economic Indexes II	0.4116	Load coefficient B1(-)	0.0368
			Station service power consumption rate B2(-)	0.0603
			Power supply coal consumption B3(-)	0.0358
			Fuel oil for ignition B4(-)	0.0444
			Fuel oil for combustion supporting B5(-)	0.0558
			Unburned carbon in flue dust B6(-)	0.0653
			Air preheater leakage rate B7(-)	0.0451
			Comprehensive water consumption rate B8(-)	0.0681
	Technical supervision indexes III	0.3124	Desulfurization system input rate C1(+)	0.0713
			Denitrification system input rate C2(+)	0.0733
			Steam water quality acceptance rate C3(+)	0.0432
			Utilization factor of thermal protection system C4(+)	0.0677
			Relay protection correct action rate C5(+)	0.0569
	Main running small indexes IV	0.1200	Service rate high pressure heater D1(+)	0.0443
			Vacuum descent rate D2(-)	0.0369
			Make-up water rate of power generation D3(-)	0.0388

The first-level index weight W_1_ = (0.1560, 0.4116, 0.3124, 0.1200)^T^ is obtained. It can be drawn from the weight of the first-level index that the reliability of TPGU is generally strong, so the reliability index is not used as the main evaluation index. The main focus is on the influence of the cost indexes such as oil, electricity and coal consumption on the TPGU and the technical supervision indexes such as desulfurization and denitration. This is also in line with the practical requirements of high efficiency, stability, energy conservation and environmental protection for TPGU. And the second-level index weight is obtained: W_2_ = (0.0279, 0.0478, 0.0393, 0.0410, 0.0368, 0.0603, 0.0358, 0.0444, 0.0558, 0.0653, 0.0451, 0.0681, 0.0713, 0.0733, 0.0432, 0.0677, 0.0569, 0.0443, 0.0369, 0.0388)T. The next step is to calculate Step5.

Step 5: Determine the weighted standardized index matrix according to (7), obtained:

$$Z=\left[\begin{array}{llll} 0.0101 & 0.0452 & 0.0500 & 0.0072\\ 0.0154 & 0.1401 & 0.0693 & 0.0090\\ 0.0111 & 0.0670 & 0.0389 & 0.0027\\ 0.0164 & 0.1467 & 0.0877 & 0.0053\\ 0.0203 & 0.1648 & 0.0735 & 0.0094 \end{array}\right]$$ (23)

Where, first-level dimensionless decision matrix Y is obtained:

$$Y=\left[\begin{array}{llll} 0.0646 & 0.1098 & 0.1600 & 0.0604\\ 0.0985 & 0.3404 & 0.2218 & 0.0748\\ 0.0713 & 0.1628 & 0.1246 & 0.0226\\ 0.1048 & 0.3563 & 0.2808 & 0.0443\\ 0.1300 & 0.4004 & 0.2351 & 0.0786 \end{array}\right]$$ (24)

Step 6: According to Eqs (8) and (9), the positive and negative ideal solutions are determined, as shown in Table 2.

**Table 2:** Positive and negative ideal solution.

First-level index	Ⅰ	Ⅱ	Ⅲ	Ⅳ
Positive ideal solution	0.0203	0.1648	0.0877	0.0094
Negative ideal solution	0.0101	0.0452	0.0389	0.0027

Step 7: The Mahalanobis Distance from each unit to the positive and negative ideal solution is obtained.

The Mahalanobis Distance requires that the number of samples is greater than the dimension of the samples. Matlab is used to calculate the relational coefficient matrix of each index according to the matrix (24), as shown in Table 3. According to Eqs (10) and (11), the Mahalanobis Distance between each unit and the positive and negative ideal solution is calculated, as shown in Table 4.

**Table 3:** Relational coefficient matrix.

index	Ⅰ	Ⅱ	Ⅲ	Ⅳ
Ⅰ	1.0000	0.9580	0.7824	0.5746
Ⅱ	0.9580	1.0000	0.8590	0.5181
Ⅲ	0.7824	0.8590	1.0000	0.4555
Ⅳ	0.5746	0.5181	0.4555	1.0000

**Table 4:** Mahalanobis distance from each TPGU to the positive and negative ideal solutions.

TPGU	*d*(*r*_*i*_, *S*^+^)	*d*(*r*_*i*_, *S*^-^)
a	2.6471	2.2038
b	3.4221	3.2610
c	3.5173	1.0996
d	2.3421	2.6452
e	1.4985	2.7576

The relational coefficient matrix of matrix (24) is obtained from Table 3. The relational coefficient between index I and index II reached 0.9580. Obviously, the direct use of Euclidean distance in TOPSIS method will lead to unreasonable evaluation results, so the Mahalanobis Distance is adopted to solve the problem.


$$\Sigma^{-1}=\left[\begin{array}{llll} 8.6480 & -0.7018 & 0.2939 & -1.4118\\ -0.7018 & 0.0716 & -0.0554 & 0.0696\\ 0.2939 & -0.0554 & 0.1114 & -0.0581\\ -1.4118 & 0.0696 & -0.0581 & 2.0074 \end{array}\right]$$ (25)

Step 8: According to Eq (7), calculate the grey relational coefficient of the *i* unit sample about the positive and negative ideal solutions of the *j* index.


$$R^{+}=\left[\begin{array}{llll} 0.8896 & 0.4079 & 0.6859 & 0.9745\\ 0.9434 & 0.7695 & 0.8175 & 0.9948\\ 0.8998 & 0.4573 & 0.6282 & 0.9249\\ 0.9543 & 0.8196 & 1.0000 & 0.9528\\ 0.9998 & 1.0000 & 0.8527 & 1.0000 \end{array}\right]$$ (26)

$$R^{-}=\left[\begin{array}{llll} 1.0000 & 1.0000 & 0.8438 & 0.9294\\ 0.9192 & 0.3865 & 0.6629 & 0.9051\\ 0.9832 & 0.7326 & 0.9995 & 0.9999\\ 0.9053 & 0.3708 & 0.5505 & 0.9581\\ 0.8545 & 0.3333 & 0.6337 & 0.8989 \end{array}\right]$$ (27)

Step 9: According to (12) and (13), the grey relational degrees between the *i* TPGU sample and the positive and negative ideal solutions are determined respectively, as shown in Table 5 below.

**Table 5:** Grey correlation degree.

TPGU	$s_{i}^{+}$	$s_{i}^{-}$
a	0.7395	0.9435
b	0.8813	0.7184
c	0.7275	0.9288
d	0.9318	0.6962
e	0.9633	0.6801

Step 10: According to Eqs (16)—(19), Mahalanobis Distance and grey correlation degree are processed dimensionless, as shown in Table 6 below.

**Table 6:** Dimensionless processing.

TPGU	*d*(*r*_*i*_, *S*^+^)	*d*(*r*_*i*_, *S*^-^)	$s_{i}^{+}$	$s_{i}^{-}$
a	0.7526	0.6758	0.7677	1.0000
b	0.9729	1.0000	0.9149	0.7614
c	1.0000	0.3372	0.7553	0.9844
d	0.6659	0.8112	0.9673	0.7379
e	0.4260	0.8456	1.0000	0.7208

Step 11: According to (20) and (21), the dimensionless Mahalanobis Distance and grey correlation degree are fused, as shown in Table 7 below.

**Table 7:** The proximity of ideal solution.

TPGU	$E_{i}^{+}$	$E_{i}^{-}$
a	0.7218	0.8763
b	0.9575	0.8672
c	0.5463	0.9922
d	0.8893	0.7019
e	0.9228	0.5734

Where, *α* = *β* = 0.5.

Step 12: The relative closeness degree of the i unit sample is calculated according to Eq (22), as shown in Table 8 below.

**Table 8:** The relative closeness degree.

TPGU	a	b	c	d	e
Relative closeness degree	0.4516	0.5247	0.3551	0.5589	0.6168

## 7. Results analysis

### 7.1. Analysis of secondary index results

**Fig 4:**
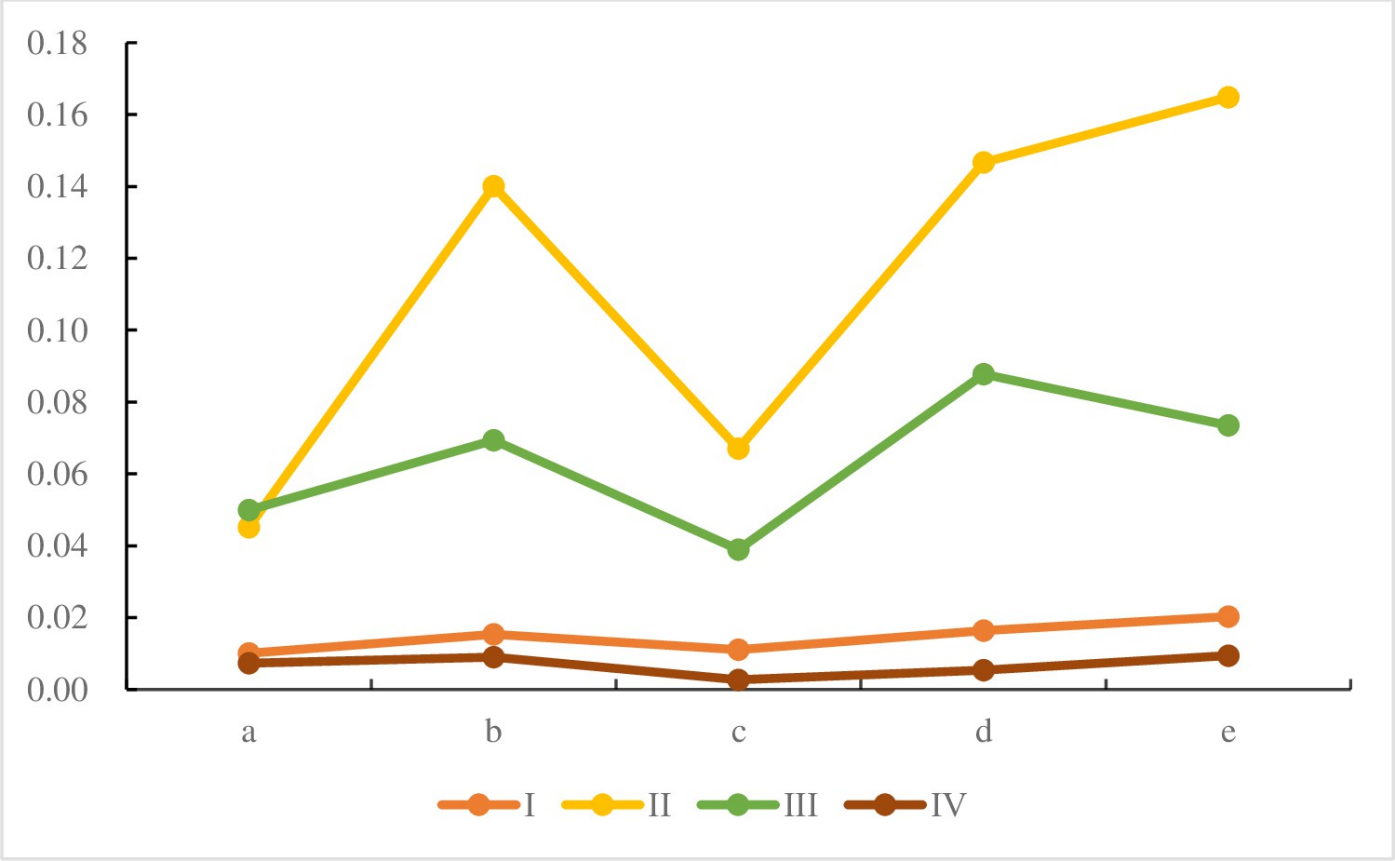
Evaluation results of second-level index.

According to the weighted standardization matrix (24) of the second-level index obtained by entropy weight method, the evaluation results of the second-level index of TPGU a, b, c, d and e can be obtained respectively, as shown in Fig 4 below

1. Reliability index I: From the reliability index alone, the order of the five units is e> d> b> c> a. The equivalent available coefficient, operating exposure rate and peak shaving coefficient belong to the benefit type index, the larger the value, the better. On the contrary, the equivalent forcing rate is the cost type index, the smaller the value, the better. According to the data in S1 Table, except for the equivalent forcing rate index, the other four indicators have a small difference, and the equivalent forcing rate is 0, so the reliability is the best.
2. Economic index II: The ranking result of economic indicators is d>e>b>a>c, and the e-unit is optimal. In S2 Table, except that the load factor is a benefit-type index, the other 7 are all cost-type indexes, and the smaller the value, the better. Two of the seven indexes about TPGU e are 0, which shows the economic superiority of TPGU e.
3. Technical supervision index III: The sorting result of this group is d>e>b>a>c. The five secondary indicators under the technical supervision index are all efficiency indicators, and the larger the value, the better. As TPGU d has three indicators with full marks, it ranks first, followed by TPGU e. Although TPGU c has full marks for two indicators, it also has two indicators of zero, so the ranking is the last. The actual analysis results are basically consistent with the ranking results.
4. Main operating indicators IV: According to Eq (24), the ranking result of this group is e>b>a>d>c. In addition to the index of service rate of high pressure heater, other two indexes are cost-type indexes. Due to the small difference in the service rate of high pressure heater, the other two indexes should be taken as the main object of analysis. Compared with other TPGUs, the difference of water supply rate of TPGU e is the largest, and the data value of vacuum falling speed is also relatively small. Therefore, TPGU e has the optimal performance of the main operating small indicators.

### 7.2. Sensitivity analysis

**Fig 5:**
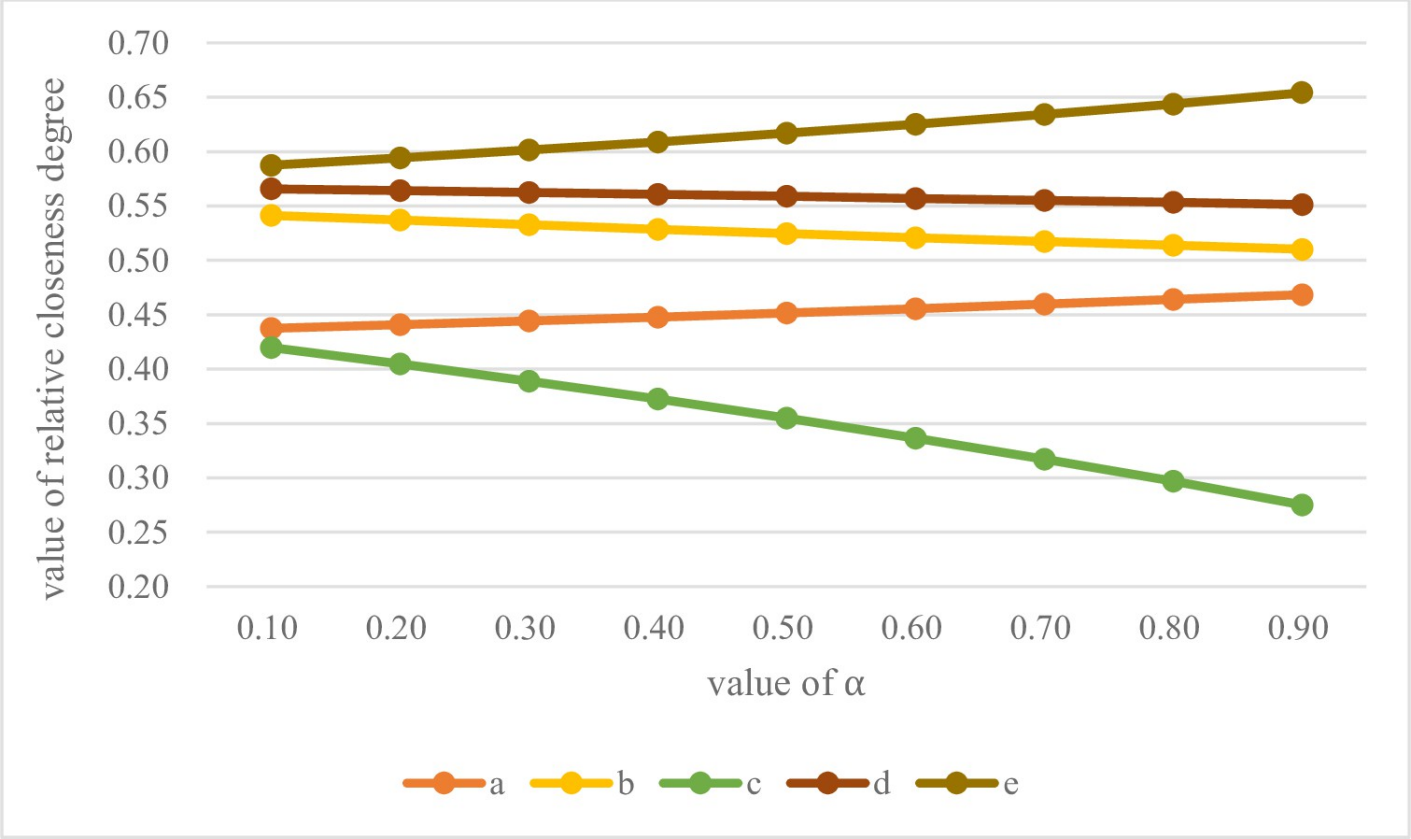
The TPGU ranking sequence trend.

In the Eqs (20)–(21), take *α* = *β* = 0.5 to obtain the relative closeness degree. In TPGU sorting process, *α* and *β* values play an indispensable role in final relative closeness degree. Generally, take *α* = *β* = 0.5, in fact, *α* and *β* can take any value between 0 and 1. In order to verify the effectiveness and robustness of the proposed results, it is necessary to analyze the sensitivities of parameters *α* and *β*. According to the values of *α* and *β* from 0 to 1, Eqs (20)–(22) is used to calculate the relative closeness degree, as shown in Table 9, and the ranking sequence trend is shown in Fig 5.

**Table 9:** The relative closeness degree based on *α* and *β* from 0 to 1.

	a	b	c	d	e
*α* = 0.1, *β* = 0.9	0.4375	0.5413	0.4198	0.5657	0.5875
*α* = 0.2, *β* = 0.8	0.4408	0.5369	0.4048	0.5640	0.5942
*α* = 0.3, *β* = 0.7	0.4443	0.5327	0.3891	0.5624	0.6013
*α* = 0.4, *β* = 0.6	0.4479	0.5287	0.3725	0.5606	0.6088
*α* = 0.5, *β* = 0.5	0.4516	0.5247	0.3551	0.5589	0.6168
*α* = 0.6, *β* = 0.4	0.4556	0.5209	0.3367	0.5570	0.6252
*α* = 0.7, *β* = 0.3	0.4597	0.5173	0.3173	0.5552	0.6342
*α* = 0.8, *β* = 0.2	0.4639	0.5137	0.2968	0.5532	0.6438
*α* = 0.9, *β* = 0.1	0.4684	0.5102	0.2752	0.5512	0.6540

As can be seen from Fig 5, the ranking order trend of the five TPGU is not affected by the value of *α* and *β*. Especially the ranking trend of *e*, which indicates that it is always the best TPGU and is not affected by *α* and *β*. The results show that the proposed Entropy—improved TOPSIS—GRA method is robust and reliable.

### 7.3. Comparative analysis

**Fig 6:**
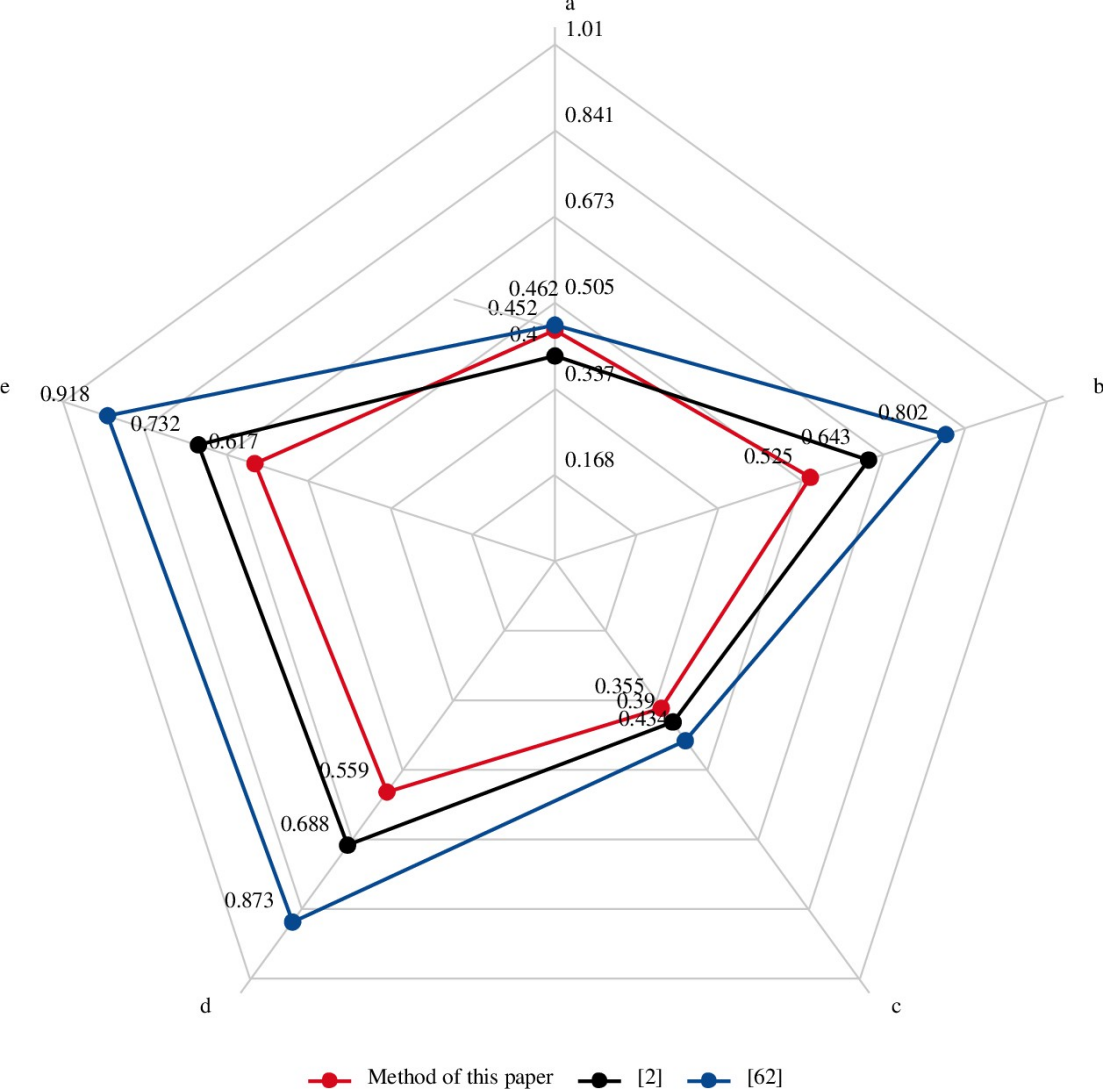
Radar chart for the three methods.

According to the relative closeness degree obtained from Table 8, the ranking of the five TPGU is *e* ≻ *d* ≻ *b* ≻ *a* ≻ *c*, which were basically consistent with Fu zhongguang [2], Qi minfang [65] and the final competition results. Final results for the methods used in this paper, the entropy weight method used by Fu zhongguang and the principal component analysis method of information entropy used by Qi minfang are shown in Table 10. The radar comparison diagram is shown in Fig 6.

**Table 10:** Final result data for the three methods.

TPGU	a	b	c	d	e
Method of this paper	0.4516	0.5247	0.3551	0.5589	0.6168
(Fu et al., 2018)	0.3998	0.6435	0.3900	0.6884	0.7317
(Qi et al., 2013)	0.4619	0.8016	0.4342	0.8728	0.9178

It can be seen from the radar chart that the results of this paper are consistent with the trend of the results obtained by the other two methods. Instead of the traditional Euclidean distance, the Mahalanobis Distance does not consider the positive and negative attributes, dimensions and units of the index. Make the algorithm easier.

### 7.4. Discussion

As can be seen from the final result radar in Fig 5, the results of all three methods are consistent. However, the numerical differences of the five groups of TPGU obtained by the Entropy-improved TOPSIS-GRA method used in this paper are small. This is due to the characteristics of the Mahalanobis Distance establishing coordinate system according to the data set, as shown in Fig 2 When the index linked between using the Euclidean distance of the conclusion is not usually reasonable. That is to say, the evaluation results of TPGU are not as different as large differences inconclusions obtained by the two methods mentioned above. Among the five sets of data, the values obtained by the three methods of unit *a* and *c* have a small difference, while the values obtained by the three methods of unit *b*, *d* and *e* have a large difference.

SWOT analysis is a widely used analysis method, which can analyze the internal resources and external environment. In order to find the development direction of TPGU, five opportunities, three threats, five strengths and five weaknesses of TPGU are summarized. Growth strategy (SO), turnaround strategy (WO), diversification strategy (ST) and defensive strategy (WT) are respectively proposed, as shown in Table 11.

**Table 11:** The SWOT analysis of TPGU.

Internal External	S (Strengths): 1. More and more attention are paid to low-carbon development. 2. The cost is low. 3. Big storage. 4. Reliability. 5. The research and development level of low-carbon thermal power was improved.	W (Weaknesses): 1. Non-renewable energy. 2. High operating costs. 3. Thermal power units have long service life. 4. Low equipment utilization. 5. Pollution of the environment.
O (Opportunities): 1. Establishment of carbon trading mechanism. 2. Establishment of ancillary services market. 3. Construction of smart power grid.4. Adjustment of the on-grid price of coal.5. Construction of uHV power grid.	1. Actively participate in carbon trading and promote the further improvement of low-carbon thermal power technology. 2. Take advantage of existing capacity and seize the opportunity of serving the electricity auxiliary market to increase power generation. 3. Make use of price advantage to increase market share.	1. Make use of the opportunity of electricity auxiliary service market of carbon trading mechanism to make up for the short board of high cost. 2. Take advantage of opportunities in the power auxiliary services market to make up for the low utilization of equipment.
T (Threats): 1. Conserve energy and reduce emissions. 2. Threat of non-fossil power generation. 3. Constraints on the coal supply chain.	1. Strengthen the management of science and technology and raise the technology level of energy conservation and emission reduction. 2. Build large-capacity, high-parameter and high-efficiency coal-fired generating units to improve the utilization rate of coal. 3. Strengthen cooperation with fuel suppliers.	1. Accelerate the upgrading of equipment to conserve energy and achieve ultra-low emissions to reduce coal consumption and pollution. 2. Optimize the cost structure, strengthen fine management, reduce operating costs.

## 8. Conclusions

In this study, the TPGU evaluation index system is explained and Entropy-improved TOPSIS—GRA method is used to phase-out TPGU in an orderly manner. As TPGU are gradually replaced by renewable energy generation, the efficiency of TPGU is gradually concerned by more and more scholars, but the research on this aspect is very limited, and the application of methods is lacking in the process of orderly phase-out. Firstly, there are uncertainties in the indicators eliminated by TPGU. Secondly, MCDM does not participate in the competition process of TPGU. Thirdly, previous research methods do not establish coordinate system for calculation from the perspective of data itself. In this paper, the TPGU evaluation index system is explained and MCDM for Entropy weight-improved TOPSIS—GRA is proposed. It provides reference for TPGU’s orderly elimination index, and the evaluation method is easier to operate. With the participation of MCDM, TPGU’s evaluation is more scientific and objective.

The Mahalanobis Distance is used to improve the classic TOPSIS in this study. And then, combined with the gray correlation, the units are ranked from the vertical and horizontal dimensions. The replacement of Euclidean distance by Mahalanobis Distance in TOPSIS method can simplify TOPSIS and expand its scope of application. The problem of index dimension inconsistency is solved well. The improved method cannot be affected by any non-singular linear transformation of the data. Taking the real data of the thermal power unit competition as the original data and according to the index system, the order of TPGU is *e* ≻ *d* ≻ *b* ≻ *a* ≻ *c*, the ranking results consistent with the results of the competition. Based on the sensitivity analysis and comparative analysis, the ranking order does not vary in general, which manifests the obtained conclusions have effectiveness and practicality. It has certain guiding significance for energy conservation, consumption reduction and optimized operation of the power plant.

The model can be used to evaluate TPGU in both vertical and horizontal dimensions, and the conclusions are more reliable, but there are still some limitations. First of all, with the increasing requirements for TPGU, the evaluation index should be updated gradually. Secondly, although the method presented in this paper has advantages over any of the previous methods, with the increase of the number of samples of TPGU, the advantages of this method in simple calculation will gradually disappear.

## Supporting information

S1 Table Reliability index (%). (DOCX)

S2 Table Economic index. (DOCX)

S3 Table Technical supervision index (%). (DOCX)

S4 Table Main operating index. (DOCX)
