# Supplementary material for: Evaluation of TPGU using entropy - improved TOPSIS - GRA method in China
Source: PLoS One. 2022 Jan 21;17(1):e0260974. doi: 10.1371/journal.pone.0260974 (PMC8782510; doi:10.1371/journal.pone.0260974)
Supplement: S1 Table — (DOCX) [file pone.0260974.s001.docx]

**S1 TABLE. Reliability index (%)**

| **PGU** | **A_1_(+)** | **A_2_(+)** | **A_3_(-)** | **A_4_(+)** |
| --- | --- | --- | --- | --- |
| a | 99.68 | 91.47 | 0.13 | 0.6498 |
| b | 91.88 | 99.35 | 1.36 | 0.773 |
| c | 85.77 | 96.46 | 1.94 | 0.8395 |
| d | 92.21 | 98.63 | 0.84 | 0.771 |
| e | 94.83 | 97.72 | 0 | 0.81 |
