# Supplementary material for: Evaluation of TPGU using entropy - improved TOPSIS - GRA method in China
Source: PLoS One. 2022 Jan 21;17(1):e0260974. doi: 10.1371/journal.pone.0260974 (PMC8782510; doi:10.1371/journal.pone.0260974)
Supplement: S2 Table — (DOCX) [file pone.0260974.s002.docx]

**S2 TABLE. Economic index**

| **PGU** | **B_1_/%(+)** | **B_2_/%(-)** | **B_3_/g·(KW·h)-1(-)** | **B_4_/t(-)** | **B_5_/t(-)** | **B_6_/%(-)** | **B_7_/%(-)** | **B_8_/m3·(MW·h)-1(-)** |
| --- | --- | --- | --- | --- | --- | --- | --- | --- |
| a | 65.3 | 8.14 | 357.43 | 668 | 705 | 6.76 | 5.86 | 0.39 |
| b | 77.23 | 5.57 | 331.45 | 114 | 215 | 0.96 | 6.06 | 0.22 |
| c | 84.1 | 6.17 | 337.85 | 762 | 739 | 0.08 | 9.95 | 3.48 |
| d | 76.3 | 5.43 | 324.21 | 217 | 0 | 0.36 | 6.61 | 0.35 |
| e | 79.82 | 5.44 | 316 | 0 | 0 | 0.17 | 4.98 | 0.3 |

Note: PGU e adopts a new type of oil-free ignition method, while other units are conventional ignition and micro-oil ignition; PGU a is a "W" type flame furnace.
