# Supplementary material for: Evaluation of TPGU using entropy - improved TOPSIS - GRA method in China
Source: PLoS One. 2022 Jan 21;17(1):e0260974. doi: 10.1371/journal.pone.0260974 (PMC8782510; doi:10.1371/journal.pone.0260974)
Supplement: S3 Table — (DOCX) [file pone.0260974.s003.docx]

**S3 TABLE. Technical supervision index (%)**

| **PGU** | **C_1_(-)** | **C_2_(+)** | **C_3_(+)** | **C_4_(+)** | **C_5_(+)** |
| --- | --- | --- | --- | --- | --- |
| a | 99.21 | 0 | 99.1 | 100 | 99.1 |
| b | 98.65 | 0 | 99.69 | 99.8 | 100 |
| c | 0 | 0 | 98.23 | 100 | 100 |
| d | 99.58 | 100 | 100 | 100 | 99.5 |
| e | 96.4 | 100 | 99.7 | 98.5 | 100 |
