# Supplementary material for: Evaluation of TPGU using entropy - improved TOPSIS - GRA method in China
Source: PLoS One. 2022 Jan 21;17(1):e0260974. doi: 10.1371/journal.pone.0260974 (PMC8782510; doi:10.1371/journal.pone.0260974)
Supplement: S4 Table — (DOCX) [file pone.0260974.s004.docx]

**S4 TABLE. Main operating index**

| **PGU** | **D_1_/%(+)** | **D_2_/Pa·min-1(-)** | **D_3_/%(-)** |
| --- | --- | --- | --- |
| a | 98.5 | 87.72 | 1.08 |
| b | 100 | 94.28 | 1.05 |
| c | 99.1 | 94.85 | 1.41 |
| d | 100 | 95.32 | 1.45 |
| e | 99.5 | 93.21 | 0.84 |
